# Supplementary material for: Machine Learning-Assisted Dual-Marker Detection in Serum Small Extracellular Vesicles for the Diagnosis and Prognosis Prediction of Non-Small Cell Lung Cancer
Source: Nanomaterials (Basel). 2022 Feb 28;12(5):809. doi: 10.3390/nano12050809 (PMC8912499; doi:10.3390/nano12050809)
Supplement: Supplementary file 1 [file nanomaterials-12-00809-s001.zip › nanomaterials-1582967-supplementary.pdf]

Supplementary information

Machine Learning-Assisted Dual-Marker Detection in Serum Small Extracellular Vesicles for the Diagnosis and Prognosis Prediction of Non-Small Cell Lung Cancer

Wenzhe Li [1,2,†], Ling Zhu [1,3,†], Kaidi Li [4], Siyuan Ye [1,3,5], Huayi Wang [1,5,6], Yadong Wang [4], Jianchao Xue [4],
Chen Wang [1,3], Shanqing Li [4], Naixin Liang [4,*] and Yanlian Yang [1,3,*]

[1] CAS Key Laboratory of Standardization and Measurement for Nanotechnology, CAS Key Laboratory of Biological Effects of Nanomaterials and Nanosafety, CAS Center for Excellence in Nanoscience, National Center for Nanoscience and Technology, Beijing 100190, China; liwenzhe@pku.edu.cn (W.L.); zhul@nanoctr.cn (L.Z.); yesy@nanoctr.cn (S.Y.); wanghuayi@cibr.ac.cn (H.W.); wangch@nanoctr.cn (C.W.)
[2] State Key Laboratory of Natural and Biomimetic Drugs, School of Pharmaceutical Sciences,
Peking University, Beijing 100871, China
[3] University of Chinese Academy of Sciences, 19 A Yuquan Rd, Shijingshan District, Beijing 100049, China
[4] Department of Thoracic Surgery, Peking Union Medical College Hospital, Chinese Academy of Medical Sciences and Peking Union Medical College, Beijing 100730, China; lkdrs@wchscu.cn (K.L.); pumc_wangyadong@student.pumc.edu.cn (Y.W.); eraser_1993@163.com (J.X.); lishanqing@pumch.cn (S.L.)
[5] Department of Chemistry, Tsinghua University, Beijing 100084, China
[6] Institute for Brain Research (CIBR), Beijing 102206, China
* Correspondence: liangnaixin@pumch.cn (N.L.); yangyl@nanoctr.cn (Y.Y.)
† These authors contributed equally to this work.

Human samples

Eligible patients were 18 years of age or older, had histologically validated stage I, II, III, or IV NSCLC, had Eastern Cooperative Oncology Group performance status score of 0 or 1 (on a 5-point scale, with higher scores indicating increasing disability; a score of 0 indicates no symptoms, and 1 mild symptoms) [1], normal organ function, adequate pulmonary function. The key exclusion criteria for NSCLC patients were previously received more than one systemic anticancer therapy, active autoimmune or infectious disease, and with clinically significant on current cancer. Demographic details of the patients are summarized in Supplementary Table S1 and S2. Inclusion criteria for healthy control donors were a negative medical history for any acute, chronic, or malignant diseases.

Table S1. Baseline Characteristics of the 33 NSCLC patients enrolled in the study.

Characteristic	Value
**Sex-number (%)**	
**Female**	17 (51.5%)
**Male**	16 (48.5%)
**Age**	
**Median — year**	56.28
**< 65 yr — number (%)**	22 (63%)
**≥ 65 yr — number (%)**	13 (37%)
**ECOG status score — no. (%)**	
**0**	21(63.6%)
**1**	12 (36.4%)
**TNM staging -number (%)**	
**I**	16 (48.5%)
**IA1**	6 (18.2%)
**IA2**	7 (21.2%)
**IA3**	1 (3.0%)
**I B**	2 (6.0%)
**II**	2 (6.0%)
**IIB**	2 (6.0%)
**III**	8 (24.2%)
**IIIA**	7 (21.2%)
**IIIB**	1 (3.0%)
**IV**	8 (22%)

Table S2. Detailed information about patients enrolled in the study.

ID	Age	Gender	TNM staging	Malignancy classification	IHC
**1**	28	Male	IA1	E/NSCLC	adenocarcinoma
**2**	58	Male	IA1		
**3**	70	Female	IA1		
**4**	45	Male	IA1		
**5**	47	Female	IA1		
**6**	66	Female	IA1		
**7**	59	Female	IA2		
**8**	70	Male	IA2		
**9**	38	Female	IA2		
**10**	45	Female	IA2		
**11**	72	Female	IA2		
**12**	83	Female	IA2		
**13**	46	Female	IA2		
**14**	56	Male	IA3		
**15**	67	Male	IB		
**16**	67	Male	IB		
**17**	49	Female	IIB	A/NSCLC	
**18**	53	Male	IIB		
**19**	46	Male	IIIA		
**20**	67	Female	IIIA		
**21**	54	Female	IIIA		
**22**	47	Female	IIIA		
**23**	46	Female	IIIA		
**24**	57	Female	IIIA		
**25**	48	Female	IIIA		
**26**	70	Male	IIIB		
**27**	40	Female	IV		
**28**	48	Male	IV		
**29**	41	Male	IV		
**30**	76	Male	IV		
**31**	51	Male	IV		
**32**	65	Male	IV		
**33**	67	Male	IV		

Table S3. Receiver operating characteristic (ROC) analysis on serum sEV EGFR, CXCR4 and combinational marker in classifying A/NSCLC (n=17) and E/NSCLC (n=16) patients, A /NSCLC patients (n=17) and HDs (n=18), as well as NSCLC patients (n=33) and HDs (n=18), respectively.

Group	Biomarker	AUC	95%CI	Sensitivity	Specificity
A/NSCLC and E/NSCLC	EGFR	0.960	89.5% -100%	94.1%,	93.8%
	CXCR4	0.842	70.9% -97.5%	76.5%	81.3%
	Combinational marker	0.963	90.4% -100%	94.1%	93.8%
A/NSCLCs and HDs	EGFR	0.977	93.8% -100%	94.1%	94.4%
	CXCR4	0.815	67.7% -95.4%	82.4%	72.2%
	Combinational marker	0.983	95.2% -100%	94.1%	94.4%
NSCLCs and HDs	EGFR	0.778	65.3% -90.3%	60.6%	88.9%
	CXCR4	0.668	51.4% -82.2%	60.6%	72.2%
	Combinational marker	0.785	66.0% -90.9%	48.5%	100%

Supplementary Figures:

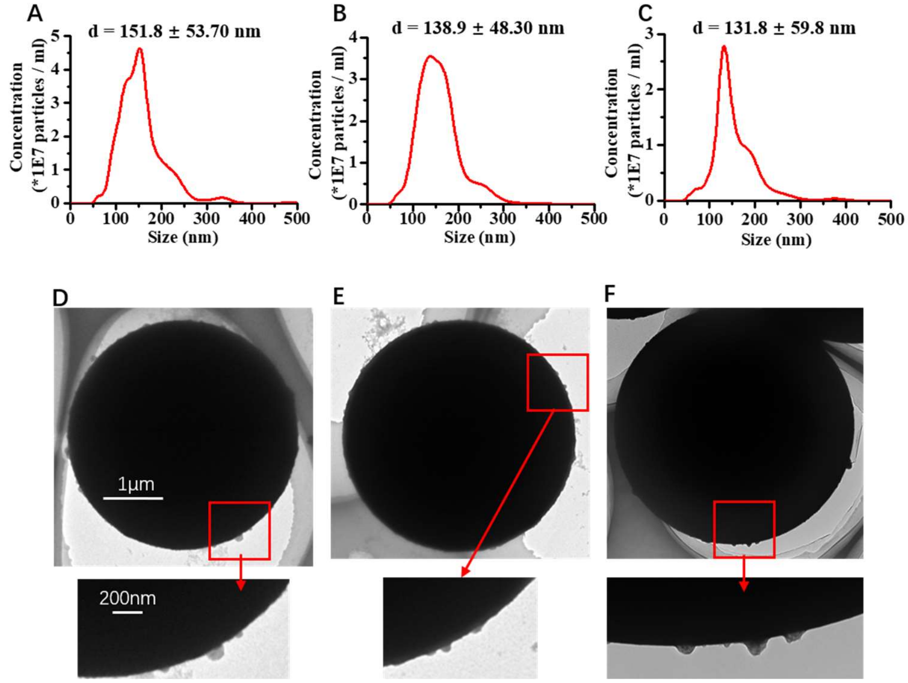

Supplementary Figure S1. Characterization and microbead enrichment of sEVs released from three non-small cell lung cancer (NSCLC) cell lines. Size distribution of sEVs released from (A) SW620, (B) H1975 and (C) H1650 cells analyzed by nanoparticle tracking analysis (NTA) and TEM images of microbead coated with sEVs released from (D) SW620, (E) H1975 and (F) H1650 cells.

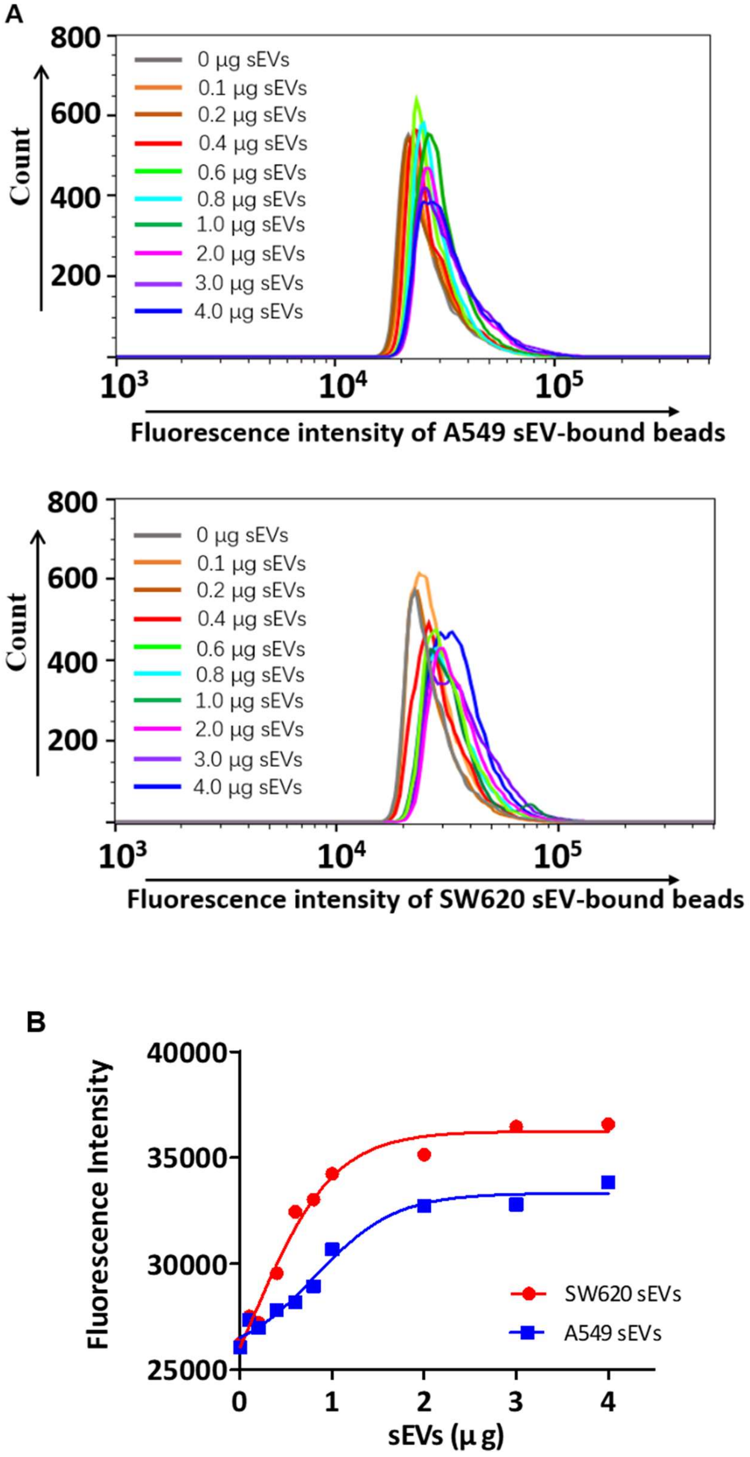

Supplementary Figure S2. Saturation assay of the binding of small extracellular vesicles (sEVs) on the aldehyde latex beads. (A) Flow cytometry analysis of the enrichment of different amounts of EVs from A549 cells (upper) and SW620 cells (lower) on 1 µL beads. (B) Saturation curve of the enrichment of sEVs on the aldehyde latex beads and the saturation concentration is about 2 µg sEVs/ µL beads.

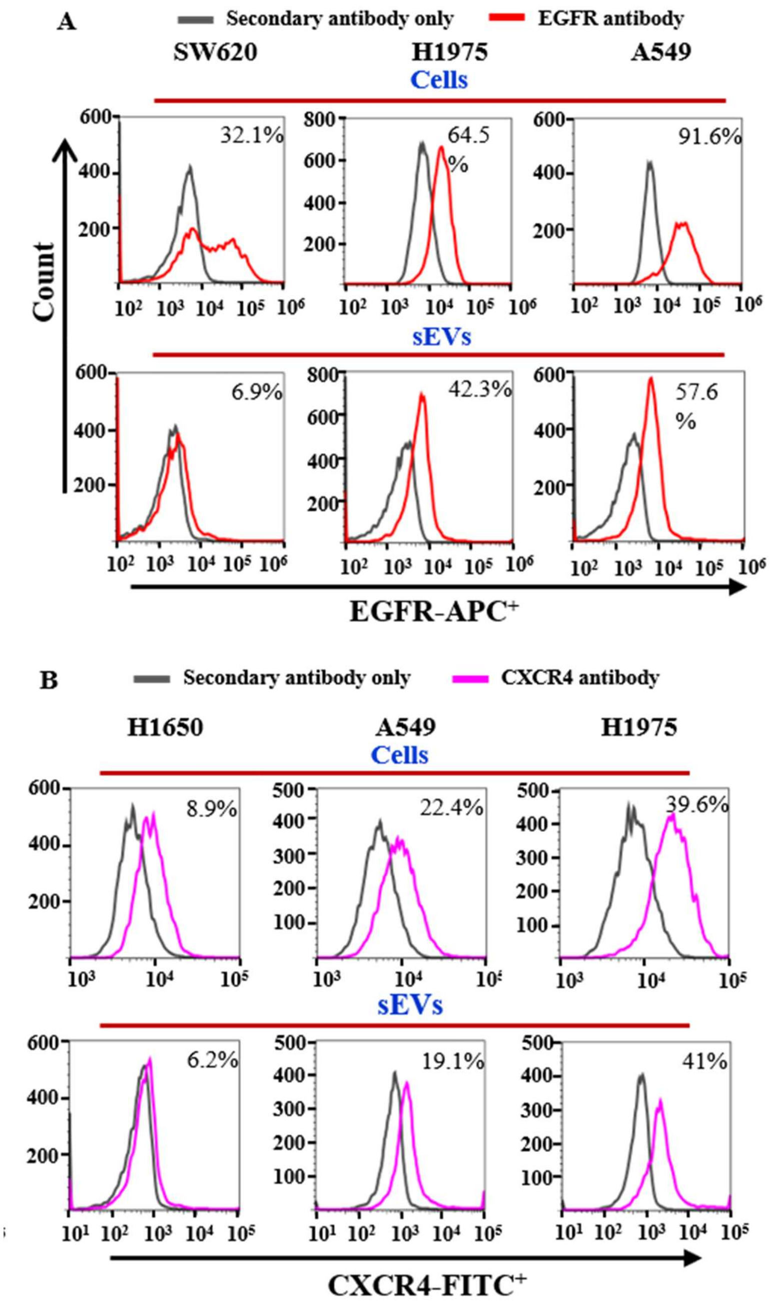

Supplementary Figure S3 Representative flow cytometry analysis of the expression of EGFR(A) or CXCR4(B) in tumor cell lines (upper lane) and tumor cell-derived EVs (lower lane).

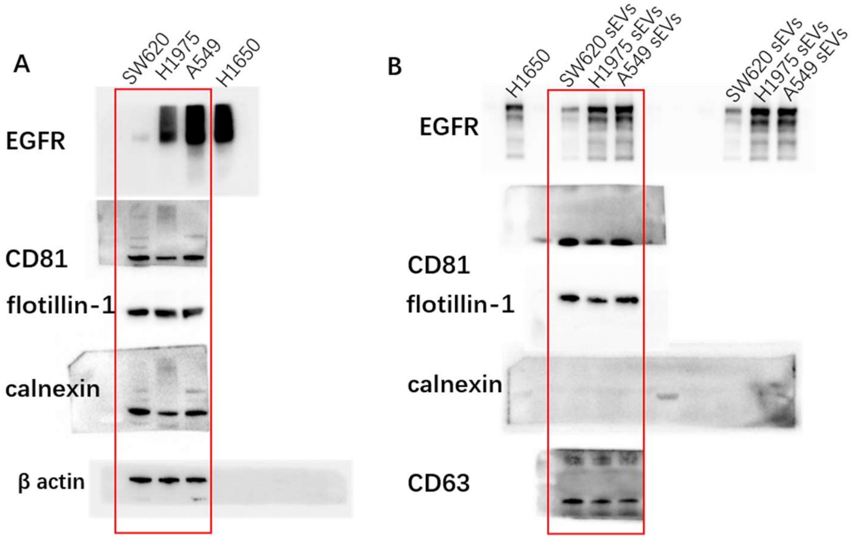

Supplementary Figure S4. Uncropped full-length blots showing the expression of EGFR in (A) cells using β actin as loading control, and (B) cell-derived sEVs using CD81, CD63 and flotillin-1 as positive controls and calnexin as negative control. **The blots in red box are used in the manuscript.**

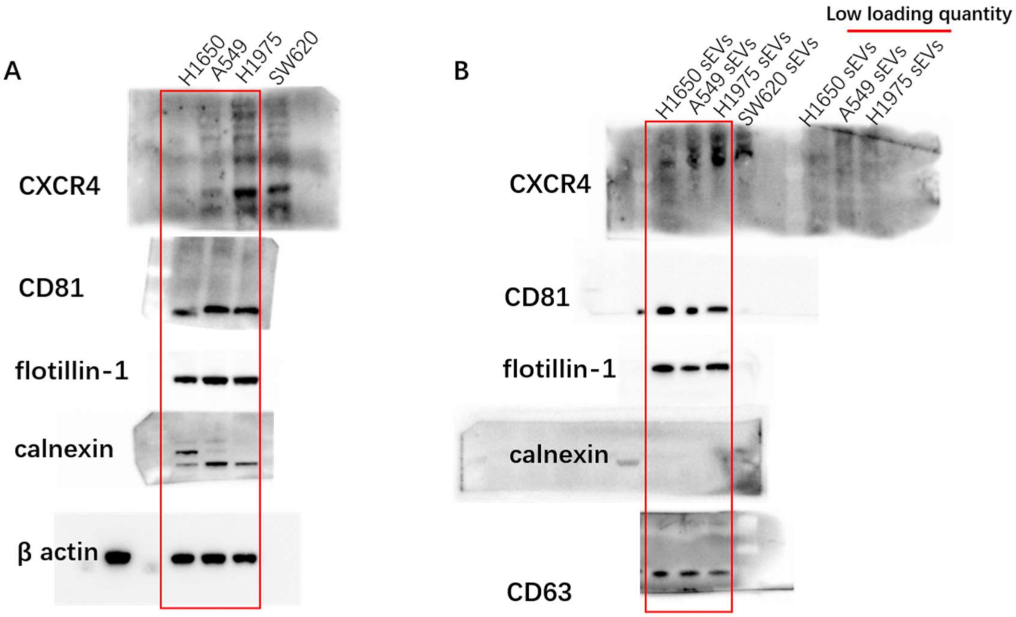

Supplementary Figure S5. Uncropped full-length blots showing the expression of CXCR4 in (A) cells using β actin as loading control, and (B) cell-derived sEVs using CD81, CD63 and flotillin-1 as positive controls and calnexin as negative control. **The blots in red box are used in the manuscript.**

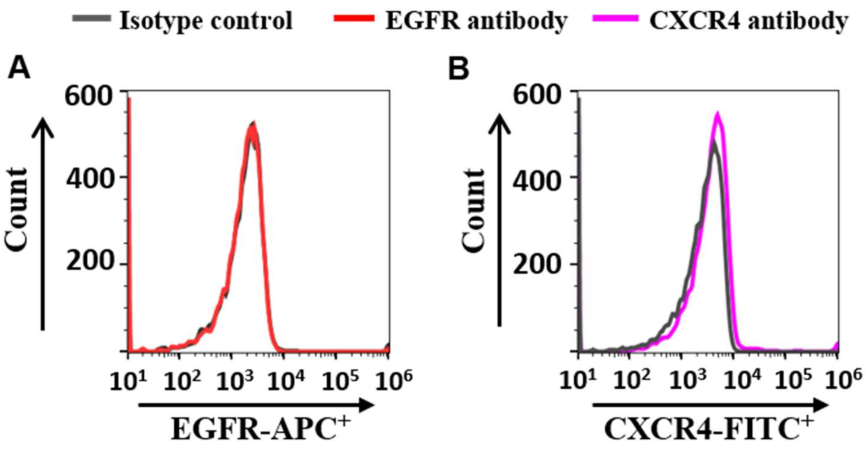

Supplementary Figure S6. Flow cytometry analysis of the primary plus secondary antibodies binding to BSA blocked beads for the expression of (A) EGFR or (B) CXCR4 in sEVs derived from A549 cell line.

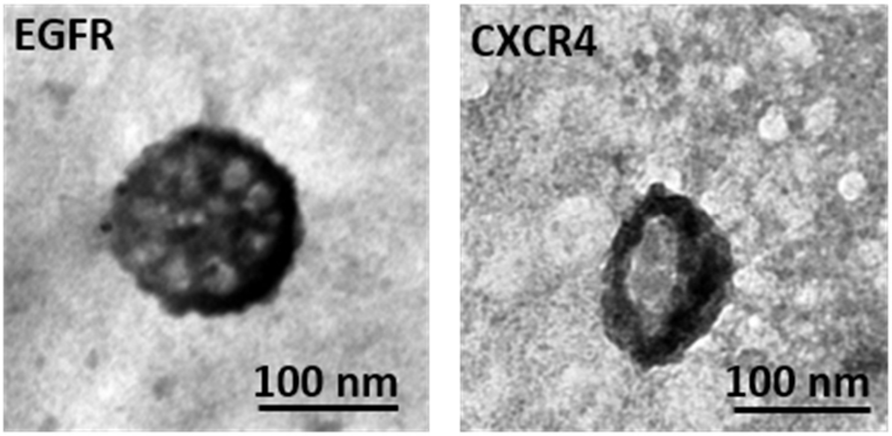

Supplementary Figure S7. Immunogold TEM images of EGFR (left) and CXCR4 (right) in sEVs from A549 cells.

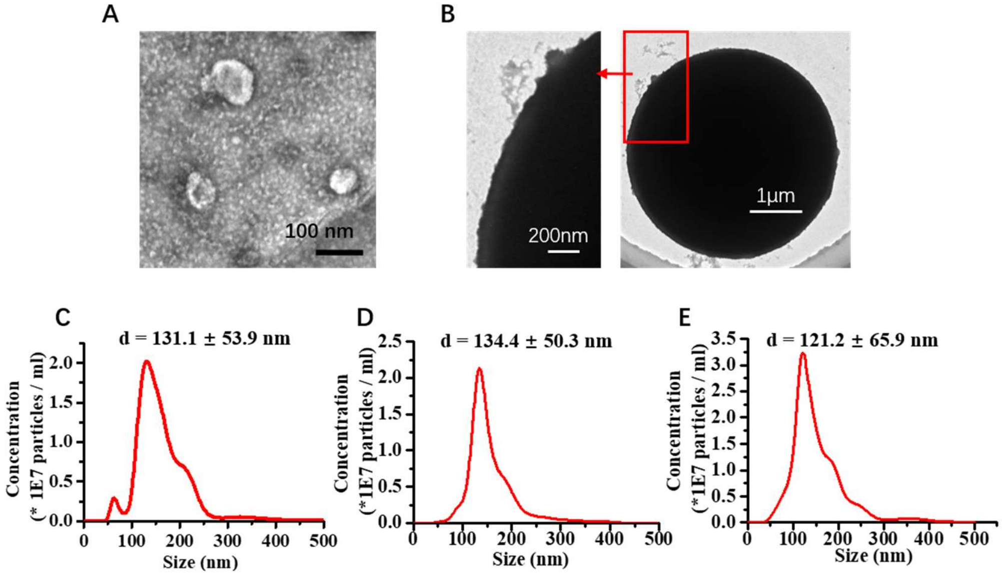

Supplementary Figure S8. Characterization of serum EVs. Transmission electron microscopy (TEM) images of (A) serum sEVs and (B) sEV-bound beads. (C-E) Size distribution of sEVs released from three samples of patient sera as analyzed by nanoparticle tracking analysis (NTA).

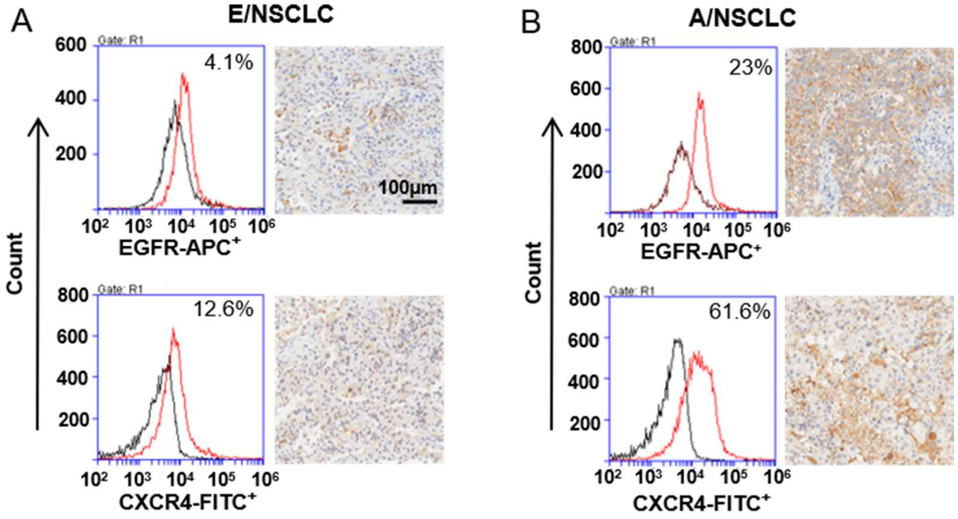

Supplementary Figure S9. The expression of EGFR or CXCR4 in serum sEVs examined by flow cytometry (left) was consistent with that in the patient-matched primary tumor tissue assessed by immunohistochemical (IHC) staining（right）in one E/NSCLC patient (A) and one A/NSCLC patient (B).

Reference

1. Oken MM, Creech RH, Tormey DC, Horton J, Davis TE, McFadden ET, et al. Toxicity and response criteria of the Eastern Cooperative Oncology Group. American Journal of Clinical Oncology. 1982; 5: 649-56.
